# PU.1 Suppresses Th2 Cytokine Expression via Silencing of GATA3 Transcription in Dendritic Cells

**DOI:** 10.1371/journal.pone.0137699

**Published:** 2015-09-11

**Authors:** Takuya Yashiro, Masato Kubo, Hideoki Ogawa, Ko Okumura, Chiharu Nishiyama

**Affiliations:** 1 Laboratory of Molecular Biology and Immunology, Department of Biological Science and Technology, Faculty of Industrial Science and Technology, Tokyo University of Science, 6-3-1 Niijuku, Katsushika-ku, Tokyo, 125–8585, Japan; 2 Atopy Research Center, Juntendo University School of Medicine, 2-1-1 Hongo, Bunkyo-ku, Tokyo, 113–8421, Japan; 3 Laboratory for Cytokine Regulation, Integrative Medical Science (IMS), RIKEN Yokohama Institute, Kanagawa, Japan; 4 Division of Molecular Pathology, Research Institute for Biomedical Science, Tokyo University of Science, 2669 Yamazaki, Noda, Chiba, 278–0022, Japan; Toho University School of Medicine, JAPAN

## Abstract

The transcription factor PU.1 is predominantly expressed in dendritic cells (DCs) and is essential for DC differentiation. Although there are several reports that PU.1 positively regulates the expression of DC-specific genes, whether PU.1 also has a suppressive effect on DCs is largely unknown. Here we demonstrate that PU.1 suppresses the expression of Th2 cytokines including IL-13 and IL-5 in bone marrow-derived DCs (BMDCs), through repression of the expression of GATA3, which is a master regulator of Th2 differentiations. When PU.1 siRNA was introduced into BMDCs, LPS-induced expression of IL-13 and IL-5 was increased along with upregulation of the constitutive expression of GATA2 and GATA3. The additional introduction of GATA3 siRNA but not of GATA2 siRNA abrogated PU.1 siRNA-mediated upregulation of IL-13 and IL-5. A chromatin immunoprecipitation assay showed that PU.1 bound to *Gata3* proximal promoter region, which is more dominant than the distal promoter in driving GATA3 transcription in DCs. The degree of histone acetylation at the *Gata3* promoter was decreased in PU.1 siRNA-introduced DCs, suggesting the involvement of PU.1 in chromatin modification of the *Gata3* promoter. Treatment with a histone deacetylase (HDAC) inhibitor, trichostatin A, increased the degree of histone H3 acetylation at the *Gata3* promoter and induced the subsequent expression of GATA3. Experiments using HDAC inhibitors and siRNAs showed that HDAC3 suppressed GATA3 expression. The recruitment of HDAC3 to the *Gata3* promoter was decreased by PU.1 knockdown. LPS-induced IL-13 expression was dramatically reduced in BMDCs generated from mice lacking the conserved GATA3 response element, termed CGRE, which is an essential site for the binding of GATA3 on the *Il-13* promoter. The degree of H3K4me3 at CGRE was significantly increased in PU.1 siRNA-transfected stimulated DCs. Our results indicate that PU.1 plays pivotal roles in DC development and function, serving not only as a transcriptional activator but also as a repressor.

## Introduction

PU.1 is a hematopoietic lineage-specific transcription factor that belongs to the Ets family. PU.1 knockout mice lack mature cells of the monocyte, neutrophil, and B and T lineages [1–4], demonstrating the requirement for this factor in myeloid and lymphoid development. It has been proposed that graded levels of PU.1 expression in hematopoietic progenitors are determinative of their lineage commitment, as high PU.1 level directs macrophage differentiation, a low level is sufficient for fetal B cell development [5, 6], and an intermediate level of PU.1 is required for granulocyte differentiation [7]. Analysis of PU.1/GFP reporter mice showed that PU.1 is expressed in all dendritic cell (DC) subsets, with myeloid DCs characteristically expressing a high amount of PU.1 and plasmacytoid DCs in comparison, expressing a low level [8].

Several previous studies including ours have demonstrated that PU.1 up-regulates the expression of the DC-characteristic genes such as class II transactivator (CIITA), CD80, CD86, and IL-12 p40 [9, 10]. PU.1 regulates gene expression by binding to canonical ETS motifs through its interaction with other transcription factors, including interferon regulatory factor 4 (IRF4), IRF8, C/EBPα and β, and c-Jun [11]. Furthermore, PU.1 is involved in chromatin remodeling by interaction with p300/CBP [12]. Although primarily recognized as a transcriptional activator, there is also emerging evidence that PU.1 can exert a repressive function. In committed myeloid progenitors, PU.1 represses transcription of osteoclast marker genes, such as *cathepsin K* and *acid phosphatase 5* [13]. Moreover, PU.1 down-regulates its target genes through epigenetic modifications including histone deacetylation and DNA methylation, by interacting with HDAC1 or Dnmt3a/b respectively in murine erythroleukemia cells [14–16].

The GATA family is comprised of six zinc-finger transcription factors, named GATA1-6. Of these factors, GATA1, 2, and 3 are indispensable for hematopoiesis. GATA3 is considered to be essential for T cell differentiation from the earliest stage of development, and is a master regulator of Th2 differentiation. Similar to other GATA family members, alternative promoters in the *Gata3* gene are used for lineage commitment. During Th2 cell development, *Gata3* transcripts include the distal exon 1a, whereas in thymocytes, the proximal exon 1b predominates. In Th2 cells, GATA3 functions as a transcriptional activator for Th2 cytokine genes such as IL-4, IL-5, and IL-13. These genes are closely linked over a 150-kb genomic region of human chromosome 5. GATA3 functions both by directly binding to Th2 cytokine loci and by chromatin remodeling of Th2 cytokine loci, thereby allowing other factors to bind more effectively [17, 18]. Similar to GATA1 and GATA2, the DNA binding activity of GATA3 is antagonized by interaction with PU.1 [19]. Thus, a Th2 cell subpopulation that expresses PU.1 exhibits lower production of Th2 cytokines [20].

In the present study, we assessed whether PU.1 serves as a transcriptional suppressor in bone marrow derived DCs (BMDCs). We demonstrate here that siRNA-mediated PU.1 knockdown resulted in the increased transcription of IL-13 and IL-5. We also show that PU.1 bound to the *Gata3* proximal promoter and repressed GATA3 expression by affecting the degree of histone acetylation of the *Gata3* promoter. We conclude that PU.1 is involved in the silencing of IL-13 and IL-5 transcription via the suppression of GATA3 expression in DCs.

## Materials and Methods

### Mice

BALB/c and C57BL/6 mice were purchased from Japan SLC (Hamamatsu, Japan). The conserved GATA3 response element (CGRE) deletion mouse, which lacks the CGRE region on the *Il-13* gene, was generated previously [21]. All animal experiments were performed according to the approved guidelines of the Institutional Review Board of Juntendo University School of Medicine, Tokyo, Japan, and of Tokyo University of Science, Tokyo, Japan. The Animal Care and Use Committees of Tokyo University of Science and of Juntendo University School of Medicine specifically approved this study.

### Cells

BMDCs were generated from the femoral and tibial bone marrow cells of female mice as described previously [22]. Cells were incubated in RPMI 1640 (Sigma-Aldrich, St Louis, MO) supplemented with 10% heat-inactivated fetal calf serum, 100 U/mL of penicillin, 100 μg/mL streptomycin, 100 μM 2-mercaptoethanol, 10 μM Minimum Essential Medium nonessential amino acid solution, and 20 ng/mL of murine GM-CSF (PeproTech, London, United Kingdom) at 37°C in a humidified atmosphere in the presence of 5% CO_2_ for 10 days.

Splenic DCs were isolated from mouse spleen as described previously [23] with slight modifications. Briefly, spleens were injected into, and incubated in RPMI 1640 containing 125 μg/ml Liberase TL (Roche Diagnostics, Mannheim, Germany), 200 μg/ml DNase I (Roche), and 10 mM HEPES at 37°C for 30 min while shaking. Single cells were prepared by passing through a 70-μm cell strainer. After centrifugation at 400 ×g for 5 min, erythrocytes were then lysed using ACK buffer.

CD11c^+^ cells were isolated by using the MACS separation system with anti-mouse CD11c MicroBeads (#130-052-001) and an autoMACS (all from Miltenyi Biotech, Tubingen, Germany).

LPS (#L3024) and trichostatin A (TSA) (#T8552) were purchased from Sigma-Aldrich. HDAC inhibitors, MS-275 (#S1053), Droxinostat (#S1422), and MC1568 (#S1484) were purchased from Selleckchem (Houston, TX).

### Small interfering RNA experiments

PU.1 siRNA (Stealth Select RNAi, Sfpi1-MSS247676), GATA2 siRNA (Gata2-MSS274487), GATA3 siRNA (Gata3-MSS274489) and control siRNA (Stealth Negative Control) were obtained from Invitrogen (Carlsbad, CA). HDAC1 siRNA (#sc-29344), HDAC3 siRNA (#sc-35539) and their control siRNA (#sc-37007) were from Santa Cruz Biotechnology (Santa Cruz, CA). A 5 μl of aliquot of 20 μmol/L siRNA was introduced into 1×10^7^ BMDCs with a Mouse Macrophage Nucleofector kit (Lonza, Basel, Switzerland) using Nucleofector II (Lonza) set at Y-001.

### Quantitative RT-PCR

The total RNA was extracted from BMDCs using an RNeasy Micro Kit (QIAGEN, Hilden, Germany) according to the manufacturer’s instructions. Complementary DNA was synthesized from 2 μg of total RNA and amplified using a ReverTra Ace qPCR RT kit (TOYOBO, Osaka, Japan). Quantitative real-time PCR was performed using an Applied Biosystems StepOne real-time PCR system (Applied Biosystems, Foster City, CA). Relative mRNA levels were obtained after normalization to the GAPDH transcript. The TaqMan ID numbers for the genes analyzed in the present study are as follows: IL-5, Mm00439646_m1; IL-13, Mm00434204_m1; PU.1, Mm00488142_m1; GATA1, Mm00484678_m1; GATA2, Mm00492300_m1; GATA3, Mm00484683_m1; GAPDH, 4352339E. For detection of the two GATA3 transcripts that are driven by different promoters, the following primers were used with the SYBR Green PCR Master Mix (#4309155, Applied Biosystems): GATA3 exon 1a forward, 5′- CCTTCAGATCTCCAGCAAGGC-3′, GATA3 exon 1b forward, 5′-GAGAGCGAGACATAGAGAGC-3′, GATA3 exon2 reverse, 5′-AGACGGTTGCTCTTCCGATC-3′.

### Measurement of IL-13 protein concentration

The concentration of IL-13 in the culture supernatant was determined using an ELISA kit (#M1300CB, R&D systems, Minneapolis, MN) according to the manufacturer’s instructions.

### Western blot analysis

The cells were lysed with RIPA buffer (50 mM Tris-HCl (pH 7.4), 150 mM NaCl, 1 mM EDTA, 1% NP-40, 0.25% sodium deoxycholate, and a protease inhibitor cocktail (Thermo Scientific, Rockford, Ill)) and were solubilized in Laemmli SDS sample buffer. Cell lysate were resolved on a 10% SDS-polyacrylamide gel (Cosmo Bio, Tokyo, Japan) and were transferred onto a polyvinylidene fluoride membrane (Bio-Rad, Hercules, CA). Anti-PU.1 (#sc-5949), anti-GATA1 (#sc-265), anti-GATA2 (#sc-9008) (all from Santa Cruz Biotechnology), anti-ß-actin (#A5441, SIGMA-Aldrich), and anti-GATA3 (#ab32858, Abcam, Cambridge, Mass) antibodies were used as the primary antibody. Peroxidase-conjugated anti-rabbit IgG (#NA934, GE Healthcare, Buckinghamshire, United Kingdom), anti-mouse IgG (#NA931) and anti-goat IgG (#705-035-147, Jackson Immuno Research, Baltimore, PA) antibodies were used as the secondary antibody, as appropriate. The membranes were soaked with Luminata Forte Western HRP Substrate (Millipore, Billerica, MA), and signals were detected with the LAS-4000 (FujiFilm, Tokyo, Japan).

### Chromatin immunoprecipitation (ChIP) assay

ChIP assays were performed as previously described [24]. Anti-PU.1 (the same clone as that used for Western blotting analysis), anti-acetyl-histone H3 (#06–599, Millipore), goat IgG (#02–6202, Invitrogen), or rabbit IgG (#02–6102, Invitrogen) were used for immunoprecipitation. Anti-HDAC3 (#ab47237), anti-H3K4me3 (#ab8580), and their control rabbit IgG (#ab46540) were purchased from abcam (Cambridge, United Kingdom). Quantitative PCR of precipitated chromosomal DNA was performed using an Applied Biosystems StepOne real-time PCR system. The nucleotide sequences of the primer sets used for quantitative PCR targeting the *GATA3* proximal promoter and CGRE on the *IL-13* promoter are described in S1 Table.

### Statistical analysis

Statistical analysis was performed using a two-tailed Student’s t-test with *p* values <0.05 considered significant.

## Results

### The effect of PU.1 knockdown on the expression of IL-13 and IL-5 in BMDCs

**Fig 1:**
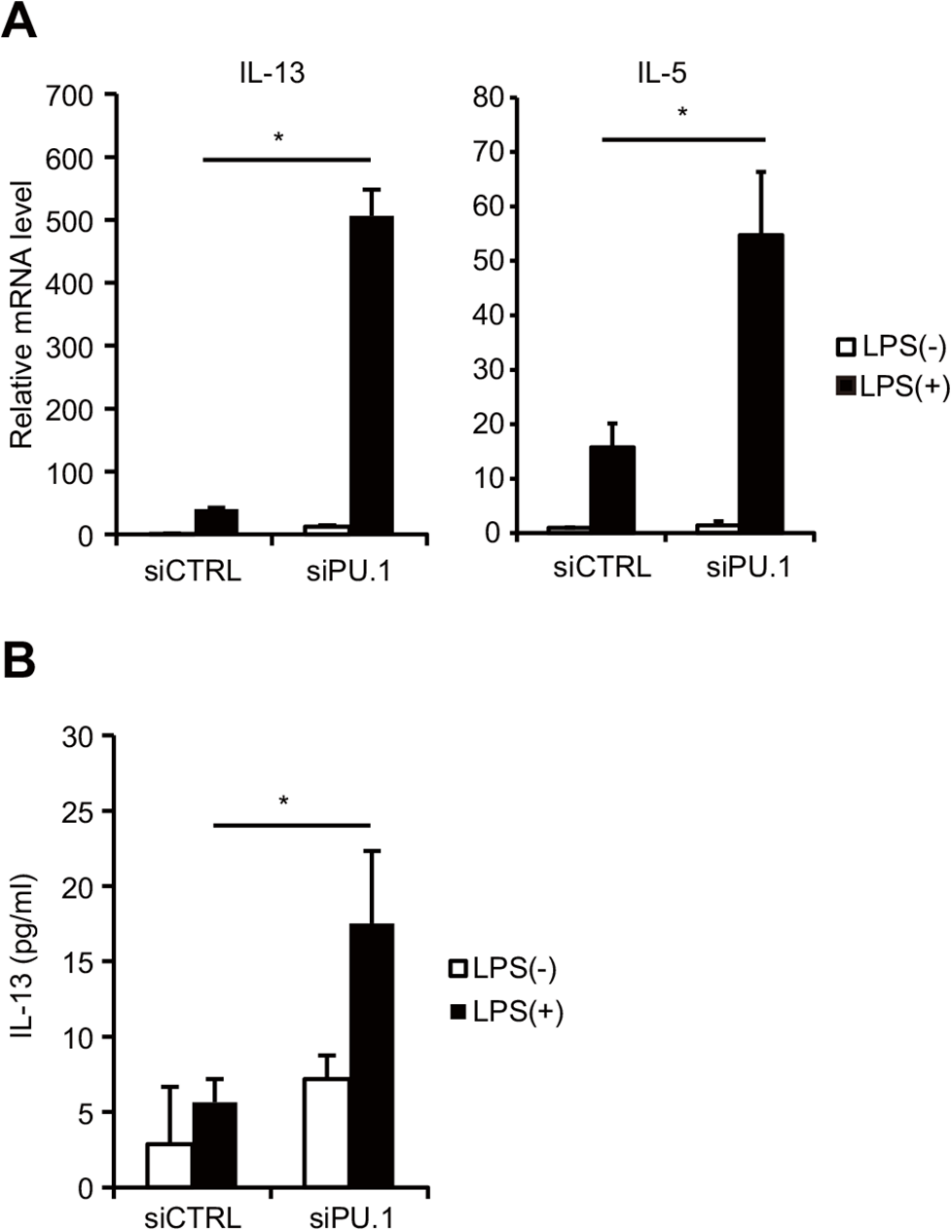
Effects of PU.1 knockdown on Th2 cytokine expression. BMDCs were transfected with either control (siCTRL) or PU.1 (siPU.1) siRNA and were then incubated for 48 h prior to stimulation with (closed bars) or without (open bars) 1 μg/ml LPS for 6 h. (A) Relative mRNA levels were determined by quantitative RT-PCR after normalization to GAPDH mRNA. Data are expressed as the ratio of the expression level of respective control siRNA-introduced cells without LPS stimulation. (B) IL-13 protein concentrations in the supernatant were measured using ELISA. All results are means ± S.E.s (*n* = 3). Similar results were obtained in three separate experiments. *, *p* < 0.05 in a two-tailed paired Student’s *t* test.

Previously, we found that PU.1 functions as a master regulator of DCs by positively regulating the expression of DC-specific genes including MHC class II (with CIITA) [10], CD80 and CD86 [9]. In contrast, the role of PU.1 in the expression of other genes that are not detected in DCs is largely unknown. During experiments to analyze the effect of PU.1 knockdown on LPS-induced TNF-α production by introduction of PU.1 siRNA into BMDCs [25], we noticed that mRNAs for Th2 cytokines were detected in PU.1 knocked-down BMDCs (unpublished observation). We therefore, hypothesized that PU.1 is involved in the DC development not only by transactivation of DC-specific genes but also by silencing DC-nonrelated genes, whose main sources are other lineages. To examine the hypothesis that PU.1 might silence the expression of DC-non-related genes, PU.1 siRNA-transfected BMDCs were analyzed as follows. We first confirmed our previous observation that PU.1 could silence the expression of IL-13, IL-5, and IL-4 in DCs. The expression level of these molecules was therefore determined using RT-PCR following LPS stimulation of control siRNA- and PU.1 siRNA-introduced DCs. LPS stimulation dramatically up-regulated IL-13 mRNA levels by approximately 600-fold in PU.1 siRNA-introduced cells (Fig 1A, left), whereas this stimulation only slightly increased the IL-13 mRNA level in control siRNA-introduced cells. ELISA of IL-13 protein levels in the culture medium showed that IL-13 production by control siRNA-introduced cells was at a low level and was not affected by LPS stimulation, but that the concentration of IL-13 protein in the culture medium of LPS-stimulated cells was significantly increased by PU.1 siRNA introduction (Fig 1B). These results suggest that PU.1 suppresses LPS-induced transcription of IL-13 mRNA and subsequent expression of the IL-13 protein. Similarly, the IL-5 mRNA level was significantly increased by PU.1 knockdown (Fig 1A, right). IL-5 and IL-4 proteins were not detected even in PU.1 siRNA-introduced cells (data not shown). Regardless, these data demonstrate that PU.1 exhibits a suppressive effect on the transcription of IL-5 and IL-13.

### GATA3 is involved in the LPS-induced IL-13 up-regulation in PU.1 siRNA-introduced BMDCs

**Fig 2:**
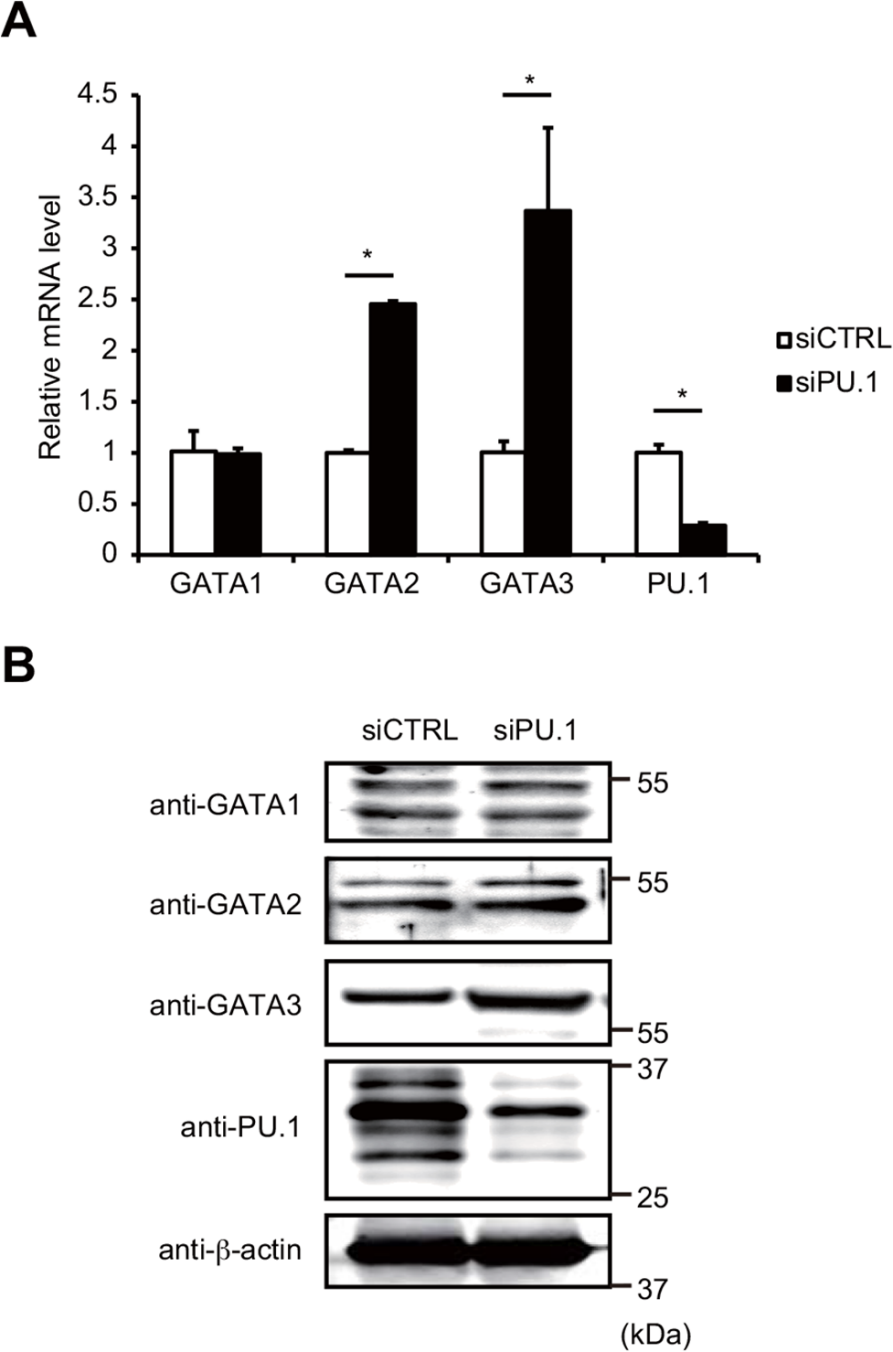
Effects of PU.1 knockdown on expression of GATAs, 1, 2, and 3. (A) BMDCs were transfected with either control (siCTRL; open bars) or PU.1 (siPU.1; closed bars) siRNA. After 48 h incubation, relative mRNA levels were determined by quantitative RT-PCR after normalization to GAPDH mRNA. Data are expressed as the ratio of the expression level of respective control siRNA-introduced cells. Results are means ± S.E.s (*n* = 3). Similar results were obtained in three separate experiments. *, *p* < 0.05 in two-tailed paired Student’s *t* test. (B) Aliquots of total proteins (15 μg/lane) of the indicated cells were subjected to SDS-PAGE and immunoblot analysis using the indicated antibodies. Similar results were obtained in three separate experiments.

Of the GATA-family proteins, GATA1, GATA2, and GATA3 are expressed in hematopoietic lineages. It is well known that GATA3 is essential for the expression of Th2 cytokines in Th2 cells [21, 26–28]. GATA1 and GATA2 are also reported to transactivate Th2 cytokine genes in mast cells [22, 29]. Although there is a report demonstrating the role of GATA1 in DCs [30], the presence and/or role of other GATA proteins in DCs have not been reported so far. Several studies have demonstrated that PU.1 suppresses the function and/or expression of GATA1, GATA2 [31], and GATA3 [20] during the development of myeloid and lymphoid lineages, respectively. We next analyzed whether PU.1 knockdown increases the expression levels of GATA1, GATA2, or GATA3, which are possible causes for the observed increase in the transcripts of IL-5 and IL-13 in PU.1-knockdown cells. Quantitative PCR analysis showed that GATA2 and GATA3 mRNA levels were increased in PU.1 siRNA-introduced DCs compared to siRNA control cells, whereas the GATA1 mRNA level in PU.1 siRNA-introduced DCs was comparable to that in control DCs (Fig 2A). Enhanced transcription of GATA2 and GATA3 was also observed using two additional PU.1 siRNAs, which were directed against different nucleotide sequences within PU.1 mRNA and were used to avoid the possible involvement of an off-target effect (data not shown). Furthermore, Western blotting of whole cell lysates of these PU.1 siRNA-transfected cells using specific antibodies indicated that the protein level of GATA3 was apparently increased by PU.1 knockdown compared to control siRNA-transfected cells, whereas the amount of GATA1 or GATA2 protein was not affected by PU.1 knockdown (Fig 2B). These results suggest that PU.1 represses the expression of GATA3 but not of GATA1 and GATA2 by BMDCs.

**Fig 3:**
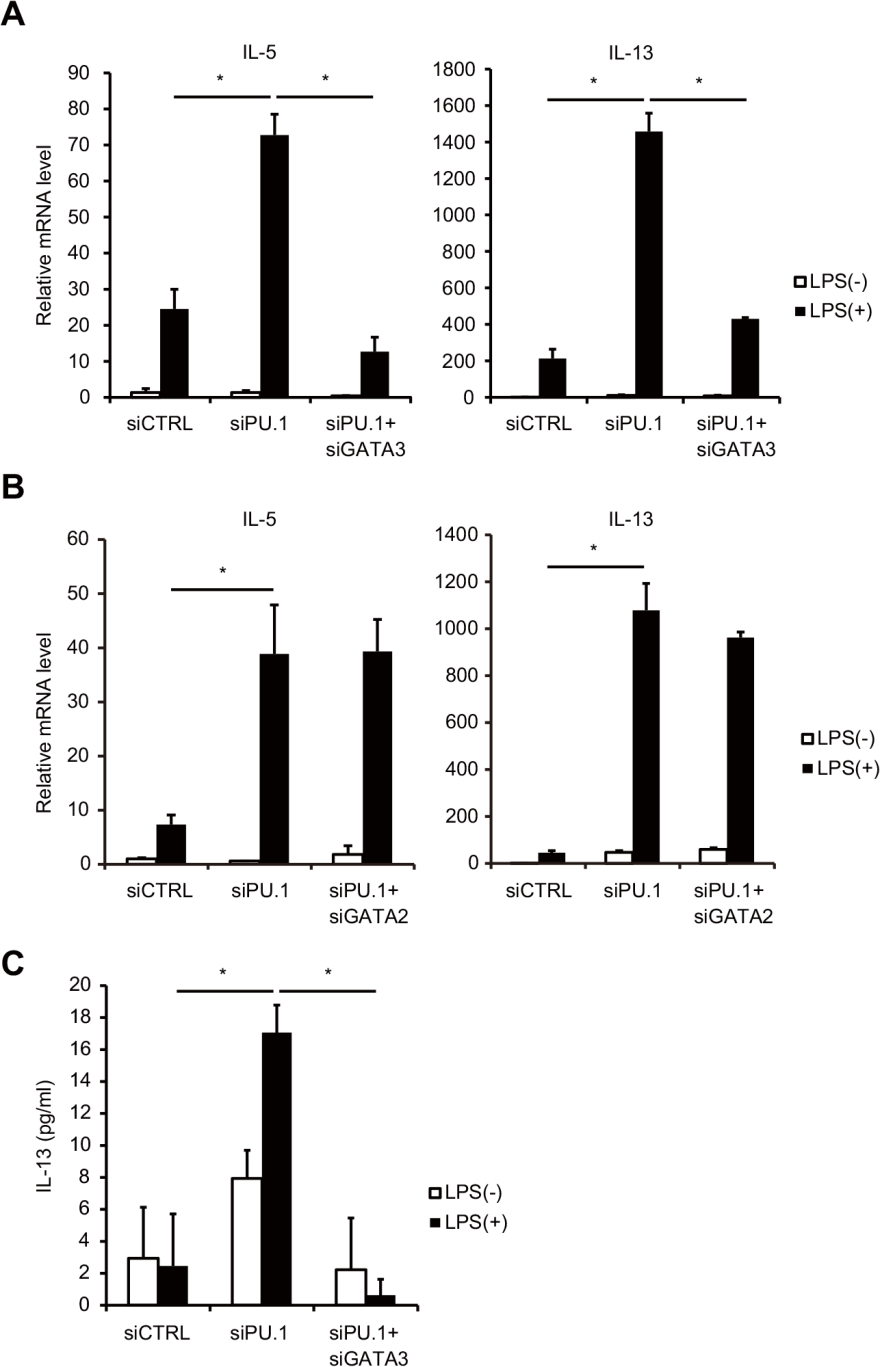
Increased expression of GATA3 is involved in the LPS-inducible IL-13 upregulation in PU.1 knockdown cells. BMDCs were transfected with the indicated siRNAs. After 48 h incubation, the cells were stimulated with (black bars) or without (white bars) of 1 μg/ml LPS for 6 h. (A, B) Relative mRNA levels of the indicated cytokines were determined by quantitative RT-PCR after normalizing to GAPDH mRNA. Data are expressed as the ratio of the expression level of respective control siRNA-introduced cells without LPS stimulation. (C) IL-13 protein concentrations in the supernatant were measured using ELISA. All results are means ± S.E.s (*n* = 3). Similar results were obtained in three separate experiments. *, *p* < 0.05 in a two-tailed paired Student’s *t* test.

To evaluate the effects of PU.1 siRNA-mediated upregulation of GATA2 and GATA3 on the PU.1 knockdown-mediated increased expression of Th2 cytokine genes, siRNA for GATA2 or GATA3 was additionally introduced into PU.1 knockdown or control siRNA-introduced cells. The increase in the mRNA levels of the Th2 cytokines IL-5 and IL-13 by PU.1 knockdown was abrogated by additional knockdown of GATA3 (Fig 3A). In contrast, the introduction of GATA2 siRNA did not affect PU.1 knockdown-mediated IL-13 or IL-5 expression (Fig 3B). Furthermore, the increase in IL-13 protein level due to PU.1 knockdown was completely abolished by additional knockdown of GATA3 (Fig 3C). These results indicated that GATA3 is involved in the LPS-mediated IL-13 production in PU.1 siRNA-introduced BMDCs. In summary, PU.1 inhibits LPS-induced IL-13 production by repressing the expression of GATA3 in BMDCs.

### PU.1 binds to the promoter region of the *Gata3* exon1b in BMDCs

**Fig 4:**
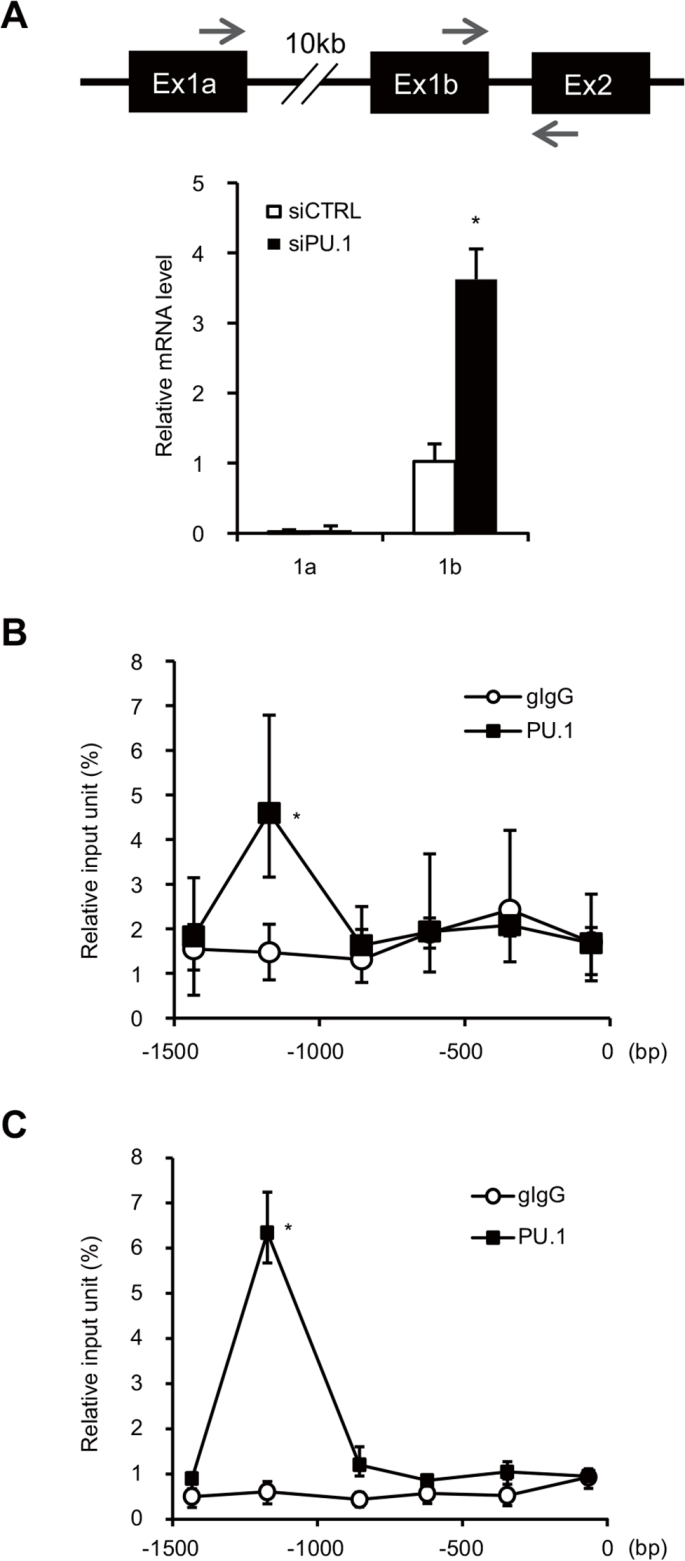
PU.1 binds to the *Gata3-1b* promoter. (A) Upper carton: Schematic drawing of the mouse *Gata3* gene. Ex, exon; the arrows over exons 1a/1b and that under exon 2, indicate forward and reverse PCR primers, respectively. Lower graph: BMDCs were transfected with either control siRNA (siCTRL) or PU.1 siRNA (siPU.1). Relative mRNA levels (1a/GAPDH or 1b/GAPDH) were determined by quantitative RT-PCR after normalization to GAPDH mRNA and are expressed as the ratio of the expression level of GATA3-1b in control siRNA-introduced cells. (B, C) Quantification of PU.1 or control goat IgG binding to the *Gata3-1b* promoter in BMDCs (B) or in splenic DCs (C) was performed using a ChIP assay with the series of primers described in S1 Table. Binding is expressed as a percentage of the input for each ChIP assay. All results are means ± S.E.s (*n* = 3). Similar results were obtained in three separate experiments. *, *p* < 0.05 in a two-tailed paired Student’s *t* test.

Previous reports demonstrated that transcription of the *Gata3* gene is controlled by two different promoters [32–35]. The distal promoter and the proximal promoter drive expression of a transcript containing the unique first exon 1a (GATA3-1a) and 1b (GATA3-1b), respectively, which splice to a common exon 2 (Fig 4A, *upper*). We measured the amount of both transcripts in PU.1 knockdown and control cells. The expression level of GATA3-1b mRNA, which was more strongly expressed than GATA3-1a mRNA in DCs, was markedly induced by PU.1 knockdown, whereas the amount of GATA3-1a mRNA was not affected by PU.1 siRNA (Fig 4A, *lower*).

To investigate whether PU.1 might bind to the GATA3-1b promoter region in DCs, we performed a ChIP assay using BMDCs and anti-PU.1 antibody. The relative levels of *Gata3-1b* chromosomal DNA associated with PU.1 were determined using a series of primers that amplify DNAs of approximately 100-bp in size (~200-bp interval) in the upstream region of GATA3-1b. As shown in Fig 4B, PU.1-specific interaction with *Gata3-1b* chromosomal DNA was detected at 1.2 kb upstream from the transcription start site of GATA3-1b. To confirm that the binding of PU.1 at this site occurs in primary DCs, a similar ChIP assay was also performed using splenic DCs. As expected, a significant amount of *Gata3-1b* chromosomal DNA bound to PU.1 was detected in ChIP assay using splenic DCs and PU.1 again bound to a site 1.2 kb upstream from the transcription start site in the *Gata3-1b* promoter (Fig 4C). Six GGAA sequences (typical Ets family-binding sequences) and six AGAA sequences (recognized by PU.1 [36]) are located in approximately 300 bp around the 1.2 kb upstream region, suggesting that PU.1 is associated with the *Gata3* proximal promoter through the direct binding to any of these PU.1 binding motifs. These results indicate that PU.1, which represses the function of the *Gata3-1b* promoter, binds to GATA3-1b promoter region on chromosomal DNA in DCs.

### Acetylation of histone H3 on the *Gata3* exon1b promoter is increased in PU.1 siRNA-introduced BMDCs

**Fig 5:**
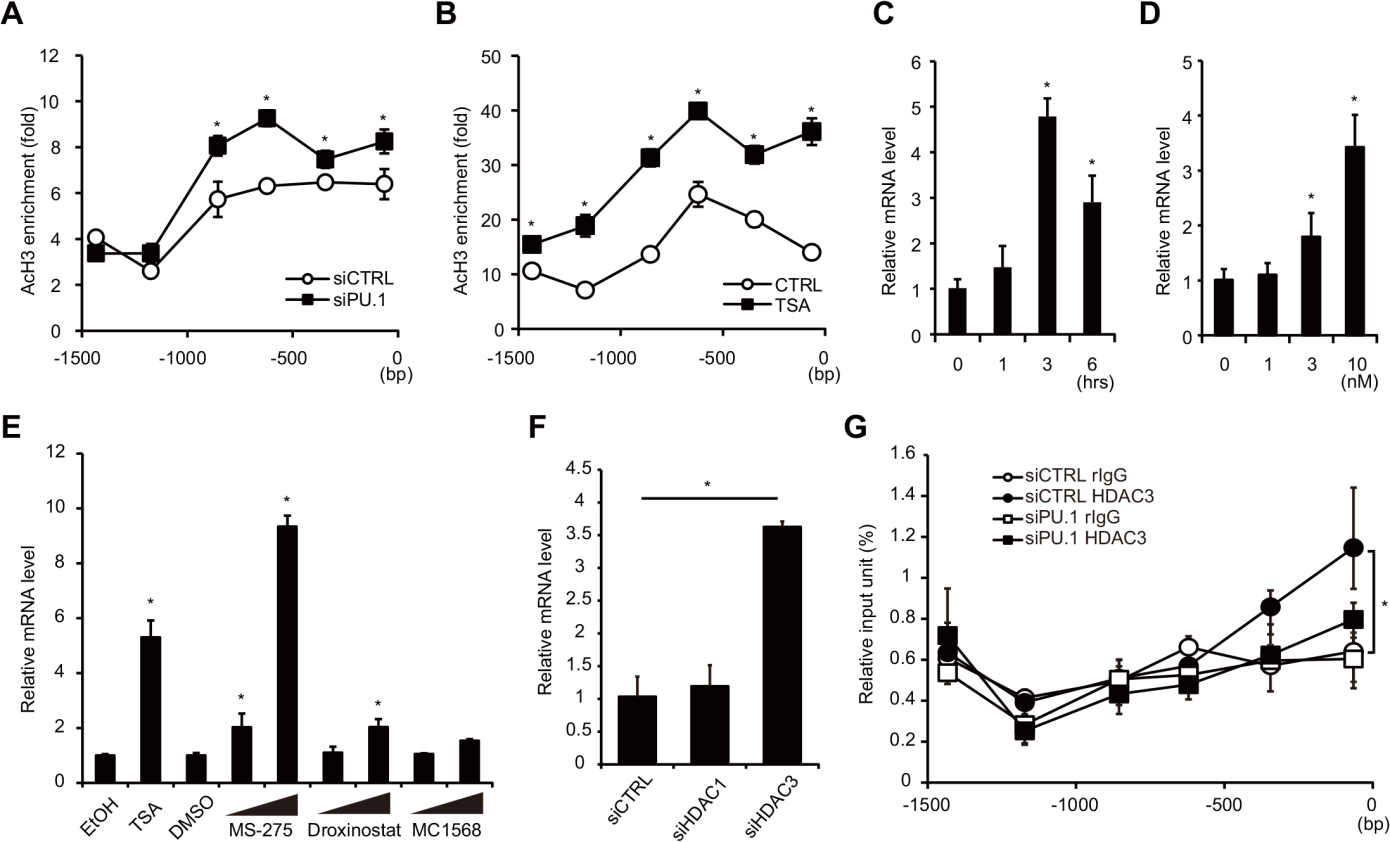
Effects of PU.1 knockdown on the histone acetylation and on HDAC recruitment at the *Gata3-1b* promoter. Histone acetylation status of the *Gata3-1b* promoter in BMDCs transfected with either PU.1 siRNA (siPU.1) or its control siRNA (siCTRL) (A), and the acetylation status in BMDCs cultured without (CTRL) or with 10 nM the histone deacetylation inhibitor trichostatin A (TSA) for 3 h (B). Quantification of acetyl-histone H3 at the *Gata3-1b* promoter was performed by ChIP assay. (C, D) GATA3 mRNA levels in BMDCs cultured with 10 nM trichostatin A for the indicated times (C) or with the indicated trichostatin A concentration for 3 h (D). Effects of HDAC inhibitors (E) and knockdown of HDACs (F) on GATA3 mRNA levels. BMDCs were treated with MS-275 (1 μM, 10 μM), Droxinostat (20 μM, 50 μM), or MC1568 (5 μM, 20 μM) for 6 h (E). Relative mRNA levels (GATA3-1b/GAPDH) were determined by quantitative RT-PCR after normalizing to GAPDH mRNA. (G) The binding degree of HDAC3 on the *Gata3-1b* promoter was analyzed by a ChIP assay. Open circles, control rabbit IgG binding in control siRNA-transfected cells; closed circles, anti-HDAC3 antibody binding in control cells; open squares, control IgG binding in PU.1 siRNA-transfected cells; closed squares, anti-HDAC3 antibody binding in PU.1 siRNA-transfected cells. All results are means ± S.E.s (*n* = 3). Similar results were obtained in three separate experiments. *, *p* < 0.05 in a two-tailed paired Student’s *t* test.

It was recently reported that PU.1 is required for chromatin modification of the IL-9 promoter in a Th subtype-specific manner, resulting in the expression of IL-9 in Th9 cells [37]. We also found that PU.1 is involved in the activation of the p1 promoter of the cofactor *Ciita* gene by increasing the acetylation state of histone H3 on the *Ciita-p1* promoter in addition to its role as a transactivator in DCs [10]. In contrast, there are several studies that have demonstrated that PU.1 functions as a transcriptional repressor by interacting with HDAC or Dnmt3a/b [14–16]. Therefore, we investigated whether PU.1 knockdown exerts an effect on the histone acetylation state of the *Gata3-1b* promoter. The levels of histone H3 acetylation at the distal region (< -1.2-kbp) of the *Gata3-1b* promoter, as analyzed using a ChIP assay with the anti-acetyl-histone H3 antibody, were not affected by PU.1 knockdown, whereas those at the proximal region (> -0.9kbp) were significantly increased (Fig 5A). This observation suggests that PU.1 contributes to enhancement of the deacetylation of histone H3 on the *Gata3-1b* promoter in BMDCs.

We next examined whether the expression of GATA3 is regulated by the histone modification state of the *Gata3-1b* promoter. For this purpose, BMDCs were incubated with trichostatin A (TSA), one of the most potent of the available histone deacetylase (HDAC) inhibitors, for 3 hours, and histone acetylation at the *Gata3-1b* promoter was then analyzed by ChIP assay using the anti-acetyl-histone H3 antibody. As shown in Fig 5B, in the presence of TSA, the levels of histone H3 acetylation were increased 2- to 3-fold at all of assessed region of the promoter, compared to control cells. Under this experimental condition, TSA significantly increased GATA3-1b mRNA levels, which peaked at 3 hours, after TSA addition (Fig 5C). This increase occurred in a TSA dose-dependent manner (Fig 5D). These results suggest that histone on the *Gata3* promoter is strongly deacetylated by HDACs in BMDCs and that inhibition of HDACs leads to transactivation of the *Gata3* promoter. The combined data show that PU.1 is involved in repression of the *Gata3* promoter in BMDCs by maintaining the chromatin status of this region in a closed conformation.

To confirm the hypothesis that PU.1 suppresses GATA3 transcription through the inhibition of HDACs, we analyzed whether the recruitment of HDACs to the *Gata3* promoter is decreased in PU.1 knockdown cells. At first, we evaluated the effects of several HDAC inhibitors on GATA3 mRNA levels to identify the HDAC(s) involved in the suppression of GATA3 expression. When BMDCs were pre-incubated with MS-275 (inhibitor for HDAC1 and HDAC3), GATA3 mRNA level in BMDCs was strikingly increased compared with that of DMSO (vehicle)-treated control cells in dose-dependent manner, whereas effects of Droxinostat (inhibitor for HDAC6 and HDAC8) and MC1568 (inhibitor for HDAC class II) were moderate and not significant, respectively (Fig 5E). This result suggests that HDAC1 and/or HDAC3 might be involved in PU.1-mediated suppression of GATA3 expression. Then, we next determined GATA3 mRNA levels in BMDCs, in which HDAC1 siRNA or HDAC3 siRNA was introduced, and found that knockdown of HDAC3 significantly increased GATA3 transcripts in BMDCs (Fig 5F). Finally, a ChIP assay was performed to examine whether HDAC3 is recruited to the *Gata3-1b* promoter in PU.1-dependent manner in BMDCs. When the amount of DNA immunoprecipitated with the anti-HDAC3 antibody was compared with that of control antibody, the specific binding of HDAC3 was detected at just upstream of transcription start site of the *Gata3-1b* promoter in control siRNA-transfected BMDCs (closed circles and open circles, Fig 5G), and the specific binding of HDAC3 on the *Gata3-1b* promoter was decreased in PU.1 siRNA-transfected cells (closed squares and open squares, Fig 5G), suggesting that HDAC3 binds to the *Gata3-1b* promoter in BMDCs in PU.1-dependent manner. These results demonstrate that HDAC3 suppresses the transcription of GATA3 and that PU.1 is involved in the recruitment of HDAC3 to the *Gata3* proximal promoter.

### The CGRE region in the *Il13* promoter is required for the PU.1 knockdown-mediated IL-13 expression

**Fig 6:**
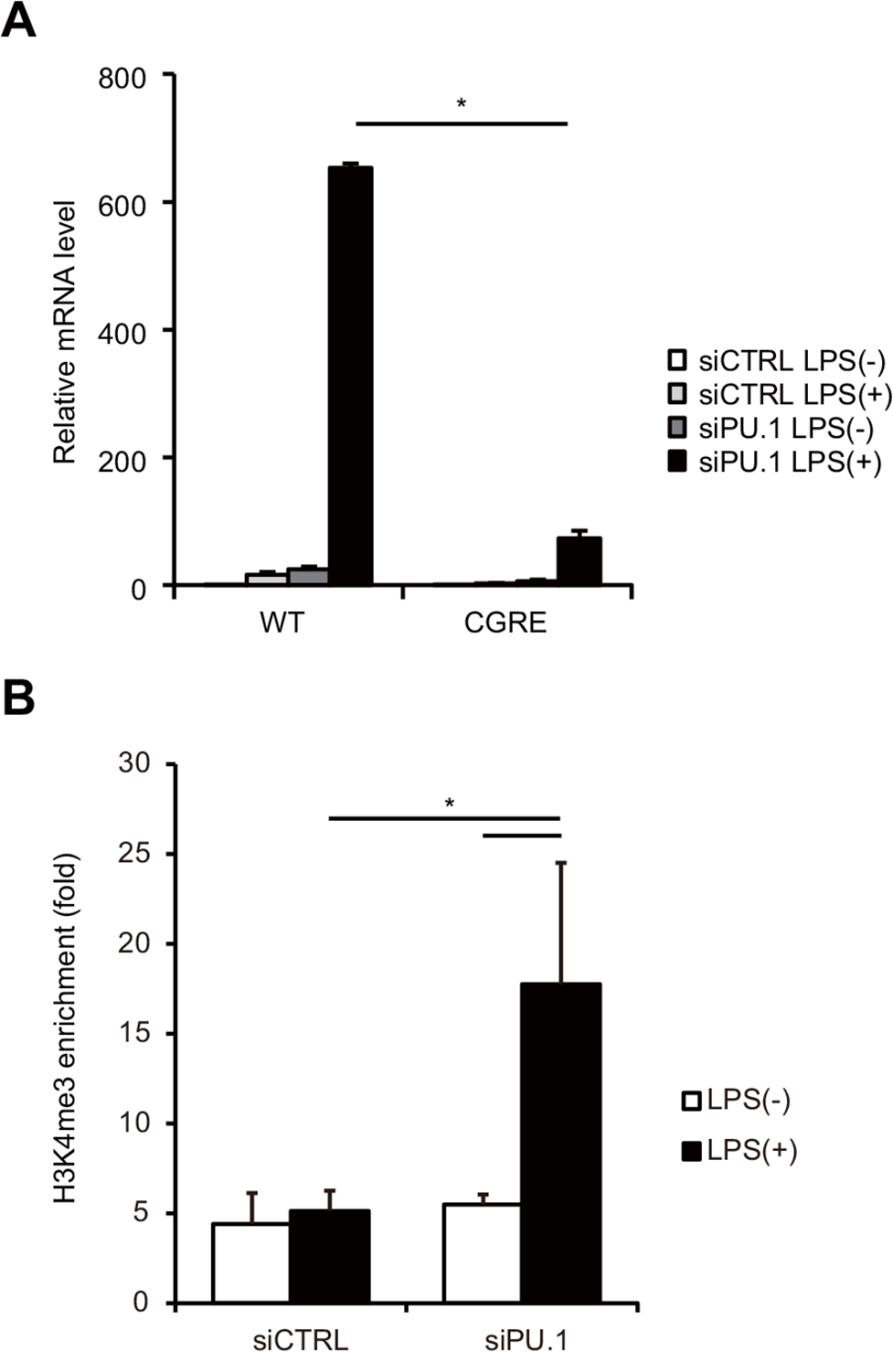
PU.1 knockdown enables GATA3 to transactivate the *Il13* promoter via affecting histone H3 modification of CGRE. (A) BMDCs from wild type (WT) or CGRE deletion (ΔCGRE) mice were transfected with either control siRNA (siCTRL) or PU.1 siRNA (siPU.1). After 48 h incubation, the cells were stimulated or not with 1 μg/ml LPS for 6 h. Relative mRNA levels (IL-13/GAPDH) were determined by quantitative RT-PCR after normalizing to GAPDH mRNA. *, *p* < 0.05 in a two-tailed paired Student’s *t* test. (B) Quantification of the H3K4me3 degree around CGRE on the *Il13* promoter was performed by a ChIP assay with anti-H3K4me3 antibody or its control antibody, and a primer set described in S1 Table. All results are means ± S.E.s (*n* = 3). Similar results were obtained in another experiment. *, *p* < 0.05 in a two-tailed paired Student’s *t* test.

CGRE, which is a conserved GATA3 response element located 1.6 kb upstream from the transcription start site of the *Il13* gene, functions as a critical *cis*-enhancing element for IL-13 expression in Th2 cells [21, 26]. In order to investigate whether CGRE is required for the PU.1 knockdown-mediated IL-13 expression, IL-13 mRNA levels in BMDCs generated from mutant mice lacking CGRE were determined. When PU.1 siRNA was introduced into BMDCs generated from WT mice, WT-BMDCs exhibited marked up-regulation of IL-13 mRNA similar to that shown in Fig 1A (Fig 6A). In contrast, the effect of PU.1 knockdown on IL-13 expression was almost abolished in CGRE-BMDCs (Fig 6A). This result demonstrates that the CGRE is necessary for the PU.1 knockdown-mediated increase of IL-13 expression.

Histone H3 of CGRE region is modified in Th2 cells but not in Th1 cells [21]. Therefore, we evaluated the effect of PU.1 knockdown on the H3 modification of CGRE by a ChIP assay using anti-H3K4me3 antibody. As shown in Fig 6B, the degree of trimethylation at Lys4 around CGRE in stimulated BMDCs was significantly increased by PU.1 siRNA transfection, suggesting that PU.1 suppresses H3 modification of CGRE in DCs.

These results indicate that GATA3 increase that results from PU.1 knockdown contributes to IL-13 expression via the H3 modification of the CGRE on the *Il13* gene.

## Discussion

Hematopoietic cell-specific transcription factor PU.1 plays an important role in the development of DCs. Although PU.1 is primarily considered as a transcriptional activator, there is increasing evidence to suggest that PU.1 also serve as a transcriptional repressor. The GATA-family consists of 6 molecules, of which GATA1, GATA2, and GATA3 are expressed in a hematopoietic cell-specific manner: GATA1 and GATA2 are essential for the erythroid/megakaryocyte development and are also involved in the development of granulocytes; GATA3 is a master regulator of Th2 cells. PU.1 induces the development toward hematopoietic lineages including monocyte and DC by suppressing the function and/or expression of GATA1 and GATA2, and induces the development toward Th9 by suppressing GATA3 [38, 39]. Therefore, GATA1, GATA2 and GATA3 have not been considered to regulate DCs, which express high levels of PU.1. Although GATA1 was recently detected in DCs and IL-4-induced upregulation of GATA1 was shown to accelerate the development of inflammatory DCs via downregulation of VDR expression [30], the presence and/or role of GATA2 or GATA3 in DCs is still unknown. In the present study, we detected GATA1, GATA2 and GATA3 in BMDCs and demonstrated that knockdown of PU.1 caused an increase in GATA2 and GATA3 levels in BMDCs. At first we expected that GATA1 and/or GATA2 could be target(s) of PU.1 in DCs, because negative cross-talk between PU.1 and GATA proteins by inhibitory effects of PU.1 (GATA1 and/or GATA2) on GATA1 and/or GATA2 (PU.1)-mediated transcriptional activation are observed in myeloid development [40–43], whereas PU.1 suppresses GATA3 in lymphoid lineages [20, 44]. Therefore, it was surprising that GATA3 as well as GATA2 was identified to be a target of PU.1 in DCs. It has been previously reported that PU.1 antagonizes GATA3 function by directly interacting with GATA3 without altering GATA3 expression levels in subpopulations of Th2 cells [20]. Considering that PU.1 silencing in BMDCs led to an increase in the mRNA levels of GATA3, and that the GATA3 transcription in DCs was driven from the proximal thymocyte-specific promoter but not from the distal Th2-specific promoter, the mechanisms by which PU.1 interferences with GATA3 functions are substantially different from those in Th2 cells.

Importantly, we showed here that PU.1 binds to the GATA3 promoter region, approximately 1.2 kb upstream from exon 1b, and that PU.1 knockdown increases the acetylation levels of histone H3 at a region downstream of its binding site. It has been recently shown that PU.1 acts as a transcriptional repressor in murine erythroleukemia cells by interacting with HDAC1 or Dnmt3a/b [14–16]. Indeed, we found that treatment with TSA enhanced the levels of GATA3-1b mRNA and histone H3 acetylation. These findings imply that PU.1 recruits co-repressor complexes including HDACs, which deacetylates surrounding histones in the GATA3 promoter region and suppresses subsequent gene expression. Among HDACs, HDAC3 is one of candidate molecules involved in PU.1-mediated suppression of GATA3 expression, because treatment with HDAC3 inhibitor and transfection of HDAC3 siRNA up-regulated GATA3 mRNA levels in DCs. Furthermore, HDAC3 was recruited to the *Gata3* promoter in PU.1-dependent manner.

We also demonstrated that the increased GATA3 resulting from PU.1 knockdown can participate in IL-13 production in response to LPS by interacting with the CGRE, which is located in its locus. Although we could not detect the expression of IL-4, another GATA3 target gene, in PU.1 knockdown cells, enhanced transcription of IL-5 was observed in addition to enhanced *Il13* transcription. DCs stimulated with LPS generally produce Th1-inducing cytokines such as IL-12, whereas they do not secrete Th2 cytokines such as IL-4, IL-5, and IL-13. Several studies have supported the idea that PU.1 regulates the gene expression of IL-12 p40 [9, 44, 45], and therefore PU.1 is postulated to be an indispensable factor for adequate cytokine production in DCs. It is well known that IL-13 is mainly produced by Th2 cells, mast cells, and basophils, in which PU.1 is expressed is little or negligible. Interestingly, retroviral expression of PU.1 in Th2 cells markedly reduces IL-13 secretion [20]. These observations suggest an inverse correlation between the expression level of PU.1 and the ability of the cells to produce IL-13, which supports our PU.1 siRNA data. Increased GATA3 that results from PU.1 knockdown might contribute to IL-13 expression via the binding to the CGRE and the modification of H3 at the CGRE. Although we could not detect GATA3-binding in the present experimental condition probably due to low levels of GATA3 in DCs (data not shown), it was confirmed that the degree of H3K4me3 of the CGRE was increased in PU.1 knockdown cells. Further detailed analysis to evaluate the involvement of PU.1 in GATA3 recruitment might reveal the mechanism of development and specific gene regulation of DCs.

In conclusion, we suggest that PU.1 represses the expression of GATA3 through PU.1 interaction with the *Gata3* proximal promoter region, resulting in a decrease in the histone acetylation status of the promoter region. Furthermore, PU.1 knockdown leading to the up-regulation of GATA3 participates in the transcription of IL-13 and IL-5. When these data are considered together with our previous studies, which showed that PU.1 positively regulates the expression of genes such as *Ciita* [10], *Cd80* and *Cd86* [9] and *Il12b* [46], PU.1 may be considered as a bifunctional regulator of DC functions; PU.1 mainly acts as a transcriptional activator for DC-characteristic gene expression, while it acts in part as a transcriptional repressor for non-characteristic gene expression. Further studies are needed to better understand the influence of PU.1 on the expression and activity of individual genes in DC functions.

## Supporting Information

S1 Table Nucleotide sequences of primers used in ChIP assays. (DOCX)
