# Supplementary material for: PU.1 Suppresses Th2 Cytokine Expression via Silencing of GATA3 Transcription in Dendritic Cells
Source: PLoS One. 2015 Sep 11;10(9):e0137699. doi: 10.1371/journal.pone.0137699 (PMC4567381; doi:10.1371/journal.pone.0137699)
Supplement: S1 Table — (DOCX) [file pone.0137699.s001.docx]

S1 Table. Nucleotide sequences of PCR primers used in ChIP assays. (related to Fig.s 4, 5, and 6)

*Gata3 ex1b* promoter

| No. |  | Sequence | Genome position (bp) |
| --- | --- | --- | --- |
| 1 | Forward | CTCCACTGGCTTCAAGGTAG | -1480~-1461 |
|  | Reverse | GGATTCCCGACAAGCCCTTTCTT | -1386~-1408 |
| 2 | Forward | GGACCCAGGCTGCAGAAA | -1201~-1184 |
|  | Reverse | CCGTCCTTTGAGTCACTGCAT | -1145~-1165 |
| 3 | Forward | CTCGGGTGAAAGCAGAATAAAGTAG | -888~-864 |
|  | Reverse | GCTGCGATAGCCTAACTTTTCTCT | -823~-846 |
| 4 | Forward | GGAAAGCAAGCAGAGACCATAAC | -651~-628 |
|  | Reverse | GGAGGACCTGGGCTTTGATT | -591~-610 |
| 5 | Forward | CAGCCGGGTTTCACTCGTA | -380~-362 |
|  | Reverse | GGCCCTTTAAATGTAGCAAAGC | -310~-331 |
| 6 | Forward | TTTGGGTTGCAGTTTCCTTG | -110~-31 |
|  | Reverse | GCAACTTAAGGAGGTTCTAG | -11~-30 |

*Il13* promoter

| Name |  | Sequence |
| --- | --- | --- |
| CGRE | Forward | GTCCTCTTATCGACCCCATC |
|  | Reverse | AAAGGCTTGGGGAAACAC |
